# Supplementary material for: Knowledge, Attitude, and Behaviour with Regard to Medication Errors in Intravenous Therapy: A Cross-Cultural Pilot Study
Source: Healthcare (Basel). 2023 Feb 3;11(3):436. doi: 10.3390/healthcare11030436 (PMC9914852; doi:10.3390/healthcare11030436)
Supplement: Supplementary file 1 [file healthcare-11-00436-s001.zip › healthcare-2076161-supplementary.pdf]

Table S1. Checklist for Reporting Of Survey Studies (CROSS)

Section/topic	Item	Item description	Reported on page #
**Title and abstract**			
Title and abstract	1a	State the word "survey" along with a commonly used term in title or abstract to introduce the study's design.	1
	1b	Provide an informative summary in the abstract, covering background, objectives, methods, findings/results, interpretation/discussion, and conclusions.	1
**Introduction**			
Background	2	Provide a background about the rationale of study, what has been previously done, and why this survey is needed.	2-3
Purpose/aim	3	Identify specific purposes, aims, goals, or objectives of the study.	2-3
**Methods**			
Study design	4	Specify the study design in the methods section with a commonly used term (e.g., cross-sectional or longitudinal).	3
Data collection methods	5a	Describe the questionnaire (e.g., number of sections, number of questions, number and names of instruments used).	3
	5b	Describe all questionnaire instruments that were used in the survey to measure particular concepts. Report target population, reported validity and reliability information, scoring/classification procedure, and reference links (if any).	3
	5c	Provide information on pretesting of the questionnaire, if performed (in the article or in an online supplement). Report the method of pretesting, number of times questionnaire was pre-tested, number and demographics of participants used for pretesting, and the level of similarity of demographics between pre-testing participants and sample population.	3
	5d	Questionnaire if possible, should be fully provided (in the article, or as appendices or as an online supplement).	3
Sample characteristics	6a	Describe the study population (i.e., background, locations, eligibility criteria for participant inclusion in survey, exclusion criteria).	3
	6b	Describe the sampling techniques used (e.g., single stage or multistage sampling, simple random sampling, stratified sampling, cluster sampling, convenience sampling). Specify the locations of sample participants whenever clustered sampling was applied.	3
	6c	Provide information on sample size, along with details of sample size calculation.	3
	6d	Describe how representative the sample is of the study population (or target population if possible), particularly for population-based surveys.	3
Survey	7a	Provide information on modes of questionnaire administration, including the type and number of contacts, the location where the survey was conducted (e.g., outpatient	2-3

administration		room or by use of online tools, such as SurveyMonkey).	
	7b	Provide information of survey's time frame, such as periods of recruitment, exposure, and follow-up days.	3
	7c	Provide information on the entry process:<br>–>For non-web-based surveys, provide approaches to minimize human error in data entry.<br>–>For web-based surveys, provide approaches to prevent "multiple participation" of participants.	3
Study preparation	8	Describe any preparation process before conducting the survey (e.g., interviewers' training process, advertising the survey).	3
Ethical considerations	9a	Provide information on ethical approval for the survey if obtained, including informed consent, institutional review board [IRB] approval, Helsinki declaration, and good clinical practice [GCP] declaration (as appropriate).	3
	9b	Provide information about survey anonymity and confidentiality and describe what mechanisms were used to protect unauthorized access.	3-4
Statistical analysis	10a	Describe statistical methods and analytical approach. Report the statistical software that was used for data analysis.	3-4
	10b	Report any modification of variables used in the analysis, along with reference (if available).	3-4
	10c	Report details about how missing data was handled. Include rate of missing items, missing data mechanism (i.e., missing completely at random [MCAR], missing at random [MAR] or missing not at random [MNAR]) and methods used to deal with missing data (e.g., multiple imputation).	3-4
	10d	State how non-response error was addressed.	3-4
	10e	For longitudinal surveys, state how loss to follow-up was addressed.	
	10f	Indicate whether any methods such as weighting of items or propensity scores have been used to adjust for non-representativeness of the sample.	
	10g	Describe any sensitivity analysis conducted.	
**Results**			
Respondent characteristics	11a	Report numbers of individuals at each stage of the study. Consider using a flow diagram, if possible.	4-9
	11b	Provide reasons for non-participation at each stage, if possible.	4-9
	11c	Report response rate, present the definition of response rate or the formula used to calculate response rate.	4-9

	11d	Provide information to define how unique visitors are determined. Report number of unique visitors along with relevant proportions (e.g., view proportion, participation proportion, completion proportion).	4-9
Descriptive results	12	Provide characteristics of study participants, as well as information on potential confounders and assessed outcomes.	4-9
	13a	Give unadjusted estimates and, if applicable, confounder-adjusted estimates along with 95% confidence intervals and p-values.	4-9
Main findings	13b	For multivariable analysis, provide information on the model building process, model fit statistics, and model assumptions (as appropriate).	4-9
	13c	Provide details about any sensitivity analysis performed. If there are considerable amount of missing data, report sensitivity analyses comparing the results of complete cases with that of the imputed dataset (if possible).	4-9
**Discussion**			
Limitations	14	Discuss the limitations of the study, considering sources of potential biases and imprecisions, such as non-representativeness of sample, study design, important uncontrolled confounders.	9-11
Interpretations	15	Give a cautious overall interpretation of results, based on potential biases and imprecisions and suggest areas for future research.	9-11
Generalizability	16	Discuss the external validity of the results.	9-11
**Other sections**			
Role of funding source	17	State whether any funding organization has had any roles in the survey's design, implementation, and analysis.	11
Conflict of interest	18	Declare any potential conflict of interest.	11
Acknowledgements	19	Provide names of organizations/persons that are acknowledged along with their contribution to the research.	11

Table S2. The trans-culturally validated questionnaire

پرسشنامه بررسی دانش، نگرش و آموزش پرستاران در طول مدیریت داروهای داخل وریدی در بخش مراقبت ویژه (ICU)

مقدمه:

خطاهای دارو در بخش مراقبت ویژه (ICU) هر روزه بیماران را در معرض خطر آسیب یا مرگ قرار می دهد. یک خطای دارویی به این صورت تعریف می شود: «هر رویداد قابل پیشگیری که ممکن است منجر به استفاده نامناسب از دارو و آسیب به بیمار شود. در حالی که این دارو تحت کنترل متخصص، بیمار یا مشتری مراقبت های بهداشتی است.» این خطاها از دیدگاه انسانی، اقتصادی و اجتماعی پرهزینه هستند. ایمنی دارو در ICU ممکن است به علت پیچیدگی شرایط بیمار، تعداد زیاد داروهای تجویز شده، تغییرات مکرر در دستورات دارویی، نیاز به دوزهای مبتنی بر وزن، پتانسیل تداخلات داروهای وریدی و محاسبه مقدار تزریق داروهای داخل وریدی به خطر بیافتد. پرستاران مسئول مدیریت و مصرف داروی بیماران هستند و مطالعات پیشین نشان داده اند که کمبود دانش، نگرش، آموزش و رفتارهای اشتباه (مثلا شستشوی نامناسب دست)، بیماران ICU را در معرض خطر قرار می دهد. بنابراین هدف مطالعه حاضر این است که تاثیر عناصر دانش، نگرش، رفتار و آموزش در جلوگیری از خطاهای دارویی در بخش های مراقبت ویژه در طول آماده سازی و مدیریت داروهای داخل وریدی را توصیف کنیم.

در این پرسشنامه از نام افراد استفاده نمی شود لذا خواهشمند است با دقت کامل و با خیال آسوده پرسشنامه را تکمیل کنید. خواهشمند است پس از مطالعه سوالات زیر پاسخ خود را در مکان تعیین شده مرقوم فرمایید.

مشخصات جمعیت شناختی:

۱. سن	۲. جنس: ☐ زن ☐ مرد	۳. کشور: ....
۴. مدرک تحصیلی:	۵. سابقه کار:	
۶. دوره ای تحت عنوان آماده سازی و مدیریت مصرف داروهای داخل وریدی IV در دوره دانشگاه گذرانده اید؟	بلی	خیر
۷. دوره ای تحت عنوان آماده سازی و مدیریت مصرف داروهای داخل وریدی IV در دوره کار گذرانده اید؟	بلی	خیر

بخش دوم: دسترسی به اطلاعات کتابشناختی به روز

عنوان	بسیار ضعیف	ضعیف	متوسط	زیاد	خیلی زیاد
۱. تا چه حد به زبان انگلیسی تسلط دارید؟	۱	۲	۳	۴	۵
۲. آیا در محیط کار به اینترنت دسترسی دارید؟				بلی	خیر
۳. آیا در محیط کار به کتابخانه (آنلاین یا فیزیکی) دسترسی دارید؟				بلی	خیر
۴. در طول هفته چند ساعت به آموزش مداوم یا ضمن خدمت اختصاص می دهید؟ ☐ کمتر از ۱ساعت در هفته ☐ بین ۱تا ۵ ساعت در هفته ☐ ۶ تا ۱۰ ساعت در هفته ☐ بیشتر از ۱۰ ساعت در هفته					

بخش سوم: آگاهی پرستاران مسئول در ICU از مصرف داروهای IV

ردیف	عنوان	کاملا مخالفم	مخالفم	نظری ندارم	موافقم	کاملا موافقم
۱	محاسبه کننده دوز داروهای داخل وریدی، خطاهای آماده سازی دارو را کاهش می دهد.	۱	۲	۳	۴	۵
۲	سیستم ثبت کامپیوتری دستورات پزشک (CPOE) خطاهای دارویی را در طول مرحله آماده سازی کاهش می دهد.	۱	۲	۳	۴	۵
۳	ارائه بسته های از قبل بسته بندی شده توسط داروخانه خطر خطاهای دارویی را کاهش می دهد.	۱	۲	۳	۴	۵
۴	در دسترس بودن دستورالعمل های آموزشی، پوستر و بروشور در بخش ها، باعث کاهش خطا می شود.	۱	۲	۳	۴	۵
۵	کمک داروساز در هنگام آماده سازی دارو باعث کاهش خطا می شود.	۱	۲	۳	۴	۵
۶	صداهای هشدار و اورژانسی بخش ممکن است باعث ایجاد حواس پرتی در آماده سازی و مدیریت دارو شود.	۱	۲	۳	۴	۵
۷	حجم کار بالا (شیفت های طولانی و متعدد) موجب خطاهای دارویی می شود.	۱	۲	۳	۴	۵

قسمت چهارم: نگرش پرستاران مسئول در ICU به استفاده از داروهای IV

ردیف	عنوان	مخالفم	نظری ندارم	موافقم
۱	آموزش های مداوم و ویژه در مورد مدیریت ایمن داروهای **IV** می تواند خطر خطاهای دارویی را کاهش دهد.	۱	۲	۳
۲	آگاهی از راه های جلوگیری از خطا و مدیریت خطر بالینی می تواند اشتباهات را در مراحل آماده سازی و مدیریت دارو کاهش دهد.	۱	۲	۳
۳	انگیزه پرستاران می تواند عملکرد حرفه ای آن ها را در طول کل روند ارائه دارو بهبود بخشد.	۱	۲	۳
۴	برای مدیریت ایمن کل فرآیند مدیریت داروهای **IV**، برخی از دستورالعمل های معتبر که با توجه به شواهد علمی موجود ایجاد شده اند، ضروری هستند.	۱	۲	۳
۵	پروتکل ها / دستورالعمل ها / اقدامات می توانند رفتار متخصصین را تحت تاثیر قرار دهند و مدیریت مناسب فرآیندهای درمانی را تضمین کنند.	۱	۲	۳
۶	مهارت های بالینی پرسنل در مورد مدیریت ایمن داروها باید مرتباً ارزیابی شوند.	۱	۲	۳
۷	خطرات دارو باید گزارش شوند تا فرصتی برای بهبود خدمات مراقبتی ایجاد شود.	۱	۲	۳

بخش پنجم: رفتار استفاده از داروهای IV توسط پرستاران مسئول در ICU

ردیف	عنوان	کاملا مخالفم	مخالفم	نظری ندارم	موافقم	کاملا موافقم
۱	شستن دست ها قبل از آماده سازی و مدیریت دارو ضروری است.	۱	۲	۳	۴	۵
۲	بررسی علائم حیاتی قبل و بعد از مصرف داروهای موثر بر عروق یا وازواکتیو (دوپامین، دابوتامین، نیتروگلیسیرین و غیره) عوارض را کاهش می دهد.	۱	۲	۳	۴	۵
۳	توجه به سرعت تزریق محلول های تجویزی داخل وریدی IV (مانند شیمی درمانی، آنتی بیوتیک ها، آمین ها، هپارین و غیره) خطاهای دارویی را کاهش می دهد.	۱	۲	۳	۴	۵
۴	پیروی از قوانین 8 Right خطاهای دارویی را کاهش می دهد.	۱	۲	۳	۴	۵
۵	قبل از تجویز، لازم است که یک بررسی مجدد برای بررسی صحت و درستی در تجویز، آماده سازی و مدیریت دارو IV انجام شود.	۱	۲	۳	۴	۵

بخش ششم: نیازهای آموزشی

ردیف	عنوان	پایین	متوسط	بالا	عالی
۱	سطح دانش خود را در مورد آماده سازی و استفاده از داروهای وریدی در چه حدی ارزیابی می کنید؟	۱	۲	۳	۴
۲	فکر می کنید که ارتقای مهارت های خود در مورد آماده سازی و تجویز داروهای وریدی تا چه حد اهمیت دارد؟	۱	۲	۳	۴
۳	پرستاران چگونه می توانند مهارت های پایه و پیشرفته خود در مورد آماده سازی و تزریق داروهای وریدی را توسعه دهند؟ (یکی از گزینه های الف تا ه را انتخاب کنید.)				

**الف**	گذاشتن کلاس های آماده سازی و مصرف دارو در دوره های آموزشی دانشگاهی و ضمن خدمت
**ب**	برگزاری کلاس آموزش ویژه در بدو ورود برای پرستاران
**ج**	شرکت مداوم در دوره های بازآموزی
**د**	انجام فعالیت های بازرسی مداوم مبنی بر بررسی صحت آماده سازی و مصرف دارو به همراه فعالیت های پژوهشی و تحقیقاتی
**ه**	سایر موارد

با این حال، اگر فکر می کنید چیزی برای افزودن وجود دارد، از فضای زیر استفاده کنید:

....
